# Development and Evaluation of the Psychometric Properties of a Brief Wisdom Development Scale

**DOI:** 10.3390/ijerph17082717

**Published:** 2020-04-15

**Authors:** Sai-fu Fung, Esther Oi-wah Chow, Chau-kiu Cheung

**Affiliations:** Department of Social and Behavioural Sciences, City University of Hong Kong, Hong Kong, China; sffung@cityu.edu.hk (S.-f.F.); ssjacky@cityu.edu.hk (C.-k.C.)

**Keywords:** assessment, geriatrics, psychometrics, wisdom, confirmatory factor analysis

## Abstract

This study was to develop an 18-item Brief Wisdom Development Scale, based on the original 66-item Wisdom Development Scale, and evaluate the psychometric properties of the proposed scale using a sample of older adults. This longitudinal study recruited 153 community-dwelling adults (mean = 72.55 years old; SD = 8.47) from older adult service centres. Using a repeated measures design, the study obtained four waves of data from the participants over 12 months. The Brief Wisdom Development Scale (BWDS) was developed based on the Stepwise Confirmatory Factor Analytical approach (SCOFA), with further verification of its factorial validity using confirmatory factor analysis (CFA). The results suggest that the BWDS comprising 18 items with a six-factor structure is comparable with its full version and possesses good psychometric properties in internal consistency, concurrent validity, and factorial validity. The BWDS provides an efficient, reliable, and valid construct to measure wisdom. The implications for research development are discussed here.

## 1. Introduction

The concept of wisdom has a long history that can be traced back to ancient civilisations. A wisdom construct has been conceptualised epistemologically and ontologically within the field of psychology in recent years [1,2]. According to Brown and Greene [3], empirical research on wisdom can be conceptualised into three categories: implicit theories of wisdom, analyses of wisdom-related performance and latent factor analyses of wisdom. They contributed to the last category by developing a Wisdom Development Scale (WDS) [3,4,5]. The focus of this study was on revising that scale by developing a short and efficient latent construct to measure wisdom

Over the past two decades, there have been several attempts to develop constructs to measure wisdom through latent factor techniques with survey methods [6,7,8,9]. Most pertinent to the present study, the WDS is comprised of 66 items with eight dimensions, namely self-knowledge, altruism, leadership, judgment, life knowledge, life skills, emotional management and willingness to learn [3,5]. The WDS is considered the longest of the constructs used to measure wisdom [10]. The scale had been recently used to study the leadership styles among secondary school principals [11].

Nevertheless, the full version of WDS may suffer from the potential issue of poor factorial validity. As such, the confirmatory factor analysis (CFA) results of WDS, in particular the CFI and TLI values (ranged from 0.67 to 0.75) reported by the original scale developers, failed to fulfil the cut-off criteria for good model fit [5]. Some scholars tried to address this issue by combining the WDS with another wisdom scale, such as Self-Assessed Wisdom (SAWS) [8,9]. In recent years, Urrutia et al. [12] suggested a 20-item measure for wisdom, based on a sample of Argentina’s older adults. Yet, their reported CFA results only suggested very marginal model fit. Combining two distinctive wisdom measures to form a new scale may have the potential danger of undermining its conceptual equivalency with the original scale.

Hence, the aims of this study were threefold. First of all, there is an urgent need to evaluate the WDS. The initial concept of the Wisdom Development Scale (WDS) was introduced in 2004 based on findings from college students [4]. Yet, within five years, the number of items and factor structure of the scale has been significantly adjusted and modified by its original authors [3,5]. As Greene and Brown [5] also urged, ‘WDS with a collegiate sample, but cross validation of those findings with other samples, as well as an examination of other types of validity, such as predictive and criterion-based studies, are needed’ (p. 290). Second, there is also a need to evaluate the WDS with different respondents and intervention programme. As suggested by Greene and Brown [5], their proposed measure of wisdom can be used to understand ‘various types of interventions and experiences’ (p. 290). To serve this purpose, this study attempts to evaluate the WDS with a clinical sample recruited from older adult service centres. Lastly, there is a scarcity of studies offering cross-cultural validation and evaluation of the WDS [13,14], and the length of the questionnaire may discourage its use by researchers and practitioners when conceiving surveys or clinical research studies [15,16]. The existing wisdom literature has vividly discussed the limitations of the existing full version of self-reported wisdom measures [1,7,13,14,17,18,19,20]. The abbreviated versions have thus been developed in recent years to address these limitations [7,12,21,22,23,24]. 

In short, this study is to critically examine the full version of WDS and develop a brief version of the WDS using the latest psychometric evaluation tools, examining its internal consistency, concurrent validity and factorial validity.

## 2. Materials and Methods

### 2.1. Participants

This longitudinal study used a repeated measures design with 153 older adults (mean = 72.55 years old; SD = 8.47) from older adult service centres in Hong Kong [25,26,27]. There were 25.5% male and 74.5% female respondents (Table 1). All of the respondents voluntarily participated in the study and possessed sufficient cognitive ability to understand and respond to the questionnaire. The research team originally recruited 157 respondents. Four incomplete questionnaires were removed from the subsequent analysis. The study was carried out in four waves, with respondents invited to fill in the questionnaire in different time frames. The initial baseline study (Study 1; *n* = 153) was conducted in June 2016. The sample was then divided into two groups: a control group (*n* = 75), and an experimental group (*n* = 78) who received narrative therapy [28,29,30,31,32]. The second wave (Study 2; *n* = 136) was conducted one month after the baseline study. Subsequently, Study 3 (*n* = 136) and Study 4 (*n* = 98) were conducted two and eight months after the initial study, respectively. The research process strictly adhered to international ethical standards with written consent obtained from all the participants. Accordingly, the participants could decide to quit the project at any time, and hence the number of samples decreased over time. The project was endorsed by the research ethics committee of the university (Ethics Approval Number: H000749).

### 2.2. Measurement

The WDS was subjected to multiple stages of development before the most commonly used format was arrived at. Brown [4] initiated the development of more than 1000 individual concepts relating to wisdom constructs, studying university students using qualitative methods. The WDS was then developed with a six-factor structure, made up of self-knowledge, interpersonal understanding, judgment, life knowledge, life skills and willingness to learn [3]. It was then further conceptualised with an eight-factor structure by splitting interpersonal understanding into altruism and leadership and extracting emotional management from life skills; the other four factors remained unchanged. Hence, the latest version of the WDS comprises 66 items with eight dimensions, namely self-knowledge (items 1–4), emotional management (items 5–9), altruism (items 10–20), inspirational engagement (items 22-31), judgment (items 32–39), life knowledge (items 40–50), life skills (items 51–61) and willingness to learn (62–66), evaluated with a seven-point Likert type scale, ranging from 1 = strongly disagree to 7 = strongly agree [5].

### 2.3. Procedure

The participants in this study were recruited from 13 older adult service centres scattered across various districts of Hong Kong. The 66 items of the WDS were translated into Chinese by two professional translators in adherence to the back-translation procedure. The translated version has been verified by the research team to ensure its semantic and conceptual equivalency as well as its sensitivity to cross-cultural differences [33,34].

The item selection process for the abbreviation version of the WDS was based on the Stepwise Confirmatory Factor Analytical approach (SCOFA) [35] and the criteria and practice stipulated in the literature on scale development and validation [36,37,38,39,40,41,42]. The following two-step procedure has been adopted. In the first step, we used the SCOFA, cross-checking the alpha if item was deleted, and McDonald’s omega to ensure that the abbreviated version is above the value of 0.70 from the data of Study 1 [43,44,45,46]. In addition, we retained at least three items per factor to ensure conceptual equivalency with the full scale and avoid the potential problem of unidentification [40]. Subsequently, CFA is conducted based on Studies 2, 3 and 4 to ensure a newly developed scale with good factorial validity. In the second step, we have to demonstrate that the proposed BWDS possesses good concurrent validity, i.e., the scale possessing strong positive correlation (> 0.70) with the full version of the WDS; and replicates the correlational direction and magnitude of the other construct-related scales reported in the wisdom literature.

To demonstrate the problem of the existing WDS, this study employed CFA to evaluate the factorial validity of the 66-item full version of the WDS and 18-item brief WDS. Due to the ordinal variables produced by the use of the Likert scale for the WDS items, diagonally weighted least squares (DWLS) were employed as the CFA estimator [47,48,49]. The CFA results were interpreted with reference to the following commonly used cut-off criteria for adequate model fit: CFI > 0.95, TLI > 0.95, RMSEA < 0.08, and SRMR < 0.08 [50,51,52]. In addition to the above criteria, the results should demonstrate acceptable model fit with χ^2^ / df ≤ 3 [53,54,55,56]. To avoid the potential for over-fitting, we conducted the CFA on the newly proposed BWDS with different datasets obtained from various waves of the study to evaluate its factorial validity [39,57,58].

The BWDS was also evaluated with psychometric testing tools: both Cronbach’s alpha [59] and corrected item-total correlation between the 18 items were used to assess the internal consistency of the reduced scale [40,60]. The McDonald’s omega, including hierarchical subscale and omega total, are also used to further evaluate the dimensionality of the scale [43,44,45,46,61].

The wisdom construct has been widely discussed in the literature as being associated positively with self-esteem, well-being and other wisdom scales [62,63,64,65,66]. Hence, the concurrent validity of the BWDS was evaluated with the following well-established measures: the Rosenberg Self-esteem (RSE) Scale comprises 10 items to measure self-esteem on a 4-point Likert-type scale ranging from 0 = strongly disagree to 3 = strongly agree. Items 3, 5, 8 9 and 10 are reversed in valence. Higher scores signify high level of self-esteem [67,68]; Personal Well-being Index (PWI) is evaluated on a 10-point Likert-type scale (0 = never; 5 = always) with 7 questions of satisfaction with specific life domains; high score indicates high level of well-being [69]; and the Self-Assessed Wisdom Scale (SAWS) comprises 40 items with a 6-point Likert-type scale (1 = strongly disagree *to* 6 = strongly agree) to measure five dimensions related to wisdom, namely emotion regulation, reminiscence, openness, experience and humour [8,9,21]. Based on the wisdom literature, the wisdom construct is expected to hold a negative correlation with depression symptoms [8,70,71]. Therefore, the Geriatric Depression Scale (GDS) was used to examine the relationship between depression and the wisdom scales. GDS is a short version consisting of 15 items with a Yes/No format (0 = No; 1 = Yes). There are five items (1, 5, 7, 11 and 13) with reversed scores. Higher scores refer to high level of depression [72,73,74,75]. The above measures, PWI (α = 0.86), RSE (α = 0.73), SAWS (α = 0.88) and GDS (α = 0.85) used in this study possess alpha values above the acceptable range. All of the above analyses were computed with the latest R (3.6.3) software (The R Foundation for Statistical Computing, Vienna, Austria) with lavaan package 0.6–5 [76] and IBM SPSS version 26.0 (International Business Machines Corporation, Armonk, NY, USA).

## 3. Results

### 3.1. Development of a Brief Wisdom Development Scale

The WDS recorded a poor factorial validity as illustrated in the results of the initial study (*n* = 153). The CFA results suggested that the full version of the WDS with a 66-item eight-factor structure failed to fulfil all of the criteria for good model fit, as χ^2^ (6445.127)/2051 = 3.14, *p* = < 0.001, SRMR = 0.10, CFI = 0.97, TLI = 0.97 and RMSEA = 0.12. These findings provide the empirical evidence to support the need to re-evaluate the factor structure and item composition of the WDS. The following sections propose a new, abbreviated version of the WDS using the above mentioned item selection procedure.

Based on the SCOFA, the newly proposed BWDS is comprised of six latent structures, including self-knowledge (1, 2 and 3), interpersonal understanding (16, 27 and 29), judgment (32, 35 and 36), life knowledge (40, 41 and 43), life skills (56, 57 and 61) and willingness to learn (63, 64 and 65). The BWDS upholds the six original factor structures advocated for in the original WDS [3,5]. The items identified above and the factor structure were further analysed with CFA. The results suggest that the newly proposed 18-item BWDS possesses good model fit, with χ^2^ (222.975)/120 = 1.86, *p* = < 0.001, SRMR = 0.06, CFI = 0.99, TLI = 0.99 and RMSEA = 0.08 (Study 1, *n* = 153). The subsequent section will further evaluate the psychometric properties of the BWDS based on longitudinal data derived from multi-wave studies (Studies 2, 3 and 4).

### 3.2. Internal Consistency

The key measures for the internal consistency of the BWDS from Study 1 (*n* = 153) are presented in Table 2. The mean score of the 18-item BWDS was 91.31 (SD = 20.11). The corrected item-total correlations for individual items in the shortened version of the WDS ranged from 0.49 to 0.76, indicating its suitability for scale construction. The scale and its six latent structures were also found to have above acceptable range in coefficient alpha (0.93), omega hierarchical (0.82) and omega total (0.96). The results also indicated that the abbreviated version is comparable with its full version (Table 3).

### 3.3. Factorial Validity

**Figure 1:**
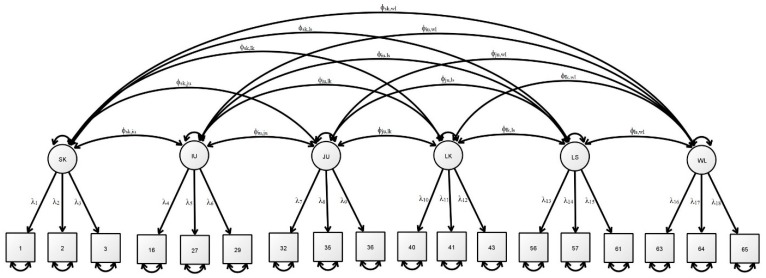
Estimated model for the 18-item Brief Wisdom Development Scale with six-factor structure.

The factorial validity of the BWDS was further evaluated with the data from Study 2 (*n* = 136), Study 3 (*n* = 136) and Study 4 (*n* = 98). The CFA results shown in Table 4 (see Figure 1 for the estimated model) suggest that all of the models fulfil the criteria for adequate model fit. The CFA results in Study 2 replicated the factor structure suggested by the Study 1 results, with χ^2^ (194.540)/120 = 1.62, SRMR = 0.06, CFI = 0.99, TLI = 0.99 and RMSEA = 0.07. The subsequent CFA results for Studies 3 and 4 also yielded similar outcomes. It is worthy of mention that the CFA results for both the control and experimental groups also supported the BWDS fulfilling all of the stringent cut-off criteria for good model fit. The control group (Combo 1) model recorded CFA results of χ^2^ (285.375)/120 = 2.38, SRMR = 0.05, CFI = 0.99, TLI = 0.99 and RMSEA = 0.07; likewise, the experimental group (Combo 2) results also suggested good model fit, returning χ^2^ (316.482)/120 = 2.64, SRMR = 0.05, CFI = 0.99, TLI = 0.99 and RMSEA = 0.08. The McDonald omega results also indicated that the proposed BWDS possesses a six-factor structure with relatively large omegaHS values, i.e., over 0.30 (*ω*_hs.SK_ = 0.45; *ω*_hs.IU_ = 0.039; *ω*_hs.JU_ = 0.67; *ω*_hs.LK_ = 0.63; *ω*_hs.LS_ = 0.68; *ω*_hs.WL_ = 0.48) [46]. Overall, these results suggest that the 18-item Brief Wisdom Development Scale with a six-factor structure possesses good factorial validity.

### 3.4. Concurrent Validity

The results of Study 1 (*n* = 153) suggest that the brief version of the wisdom construct scale and its latent structures are comparable with its full version (Table 5). As such, the 66-item full version of the WDS correlated significantly with the 18-item abbreviated version of the WDS, with *r* = 0.95, *p* < 0.001.

The BWDS also replicated the correlational direction and magnitude of other wisdom-related measurements of well-being, self-esteem, depression and other wisdom constructs. The BWDS had significant moderate to strong positive correlations with the PWI (*r* = 0.43, *p* < 0.001), RSE (*r* = 0.45, *p* < 0.001) and SAWS (*r* = 0.75, *p* < 0.001), as illustrated in Table 6. The 18-item BWDS also exhibited a significant moderate negative correlation with GDS (*r* = −0.43, *p* < 0.001). Hence, the results indicate that the BWDS holds good concurrent validity with other well-established wisdom construct–related scales.

## 4. Discussion

The empirical data support and justify the development of the 18-item brief version of the Wisdom Development Scale. The CFA results of the 66-item full version of the WDS were similar to those reported by Greene and Brown [5], which failed to fulfil the current standard criteria for adequate model fit. In general, the proposed BWDS possesses good psychometric properties with the alpha coefficient of 0.93 similar to that reported for the 66-item full version of the WDS (α = 0.95) [3]. We also found that the shortened version is comparable with the full version, as reflected by the following metrics. In view of the concurrent validity, both scales hold the same correlational direction and magnitude in relation to the wisdom-related constructs, such as well-being, self-esteem and depression. The BWDS also has a very strong significantly positive correlation (0.95) with its full version. The total score of BWDS in Studies 1, 2, 3 and 4 are consistent, with x = 91.31 (SD = 20.1), 92.72 (SD = 18.27), 93.29 (SD = 17.69), and 92.78 (SD = 19.34), respectively.

The six-factor latent structure identified in the BWDS echoes the conceptual factor structure of the original scale’s theoretical assumptions, i.e., self-knowledge, interpersonal understanding, judgment, life knowledge, life skills and willingness to learn [3,5]. The scale developers subsequently added the seventh and eighth factors into the scale. However, according to Brown and Greene [3], the eight-factor structure in their exploratory factor analysis results only yielded 40% of the total variance. They also admitted that some ‘items will be set aside for future research into why they loaded together, but given that their grouping did not fit with the theoretical framework of the study’ (p. 11). The two additional factors, emotional management and altruism, were split into multiple subscales from interpersonal understanding and life skills, respectively, due to the complicated factor structure. Their reported CFA results also indicated a poor model fit for the eight-factor structure. The present study offers a solution to these issues, by proposing an abbreviated version of the WDS with an item selection strategy suggested by Schroeders, Wilhelm and Olaru [35]. The advantage of SCOFA can compile the short version with high reliability, but may have issue with validity. To avoid this problem, this study involved a two-step procedure for the item selection process to develop the abbreviated version of the WDS. The results demonstrated that the shortened version of WDS possesses good psychometric properties, including internal consistency and convergent validity. Furthermore, the CFA results support the 18-item BWDS with six latent factors as fulfilling all of the latest requirements for good model fit.

This study may have the following limitations. First, the small sample size may hinder the reliability of the findings, and could explain why the RMSEA values in Study 4 (*n* = 98) only yielded a marginally adequate value of 0.09 (95% CI). With a 90% confidence interval, the RMSEA values are within the threshold of the acceptable range. The small sample size is due to the difficulties involved in recruiting a large number of older adults from the service centres. We attempted to use two measures to alleviate the problem. This study uses DWLS as the CFA estimator. The simulation studies suggested that this estimator works well with the model with small sample size (N > 50) [47,77,78,79,80]. The research design of this study may also counteracts this limitation by using a longitudinal study with a repeated measures design [81]. Consequently, the CFA results at different time points, pretests and posttests after interventions all indicated a good model fit for the BWDS. One may suggest further examination of the measurement invariance of the scale. Yet there are still many outstanding ‘methodological issues surrounding the measurement invariance testing’ (p. 78) which may be beyond the scope of this paper [82]. Second, the demographic background of the respondents may limit the variance of the data. The older adults participating in this study have an average age of over 70 and are settled in older adult service centres on a long-term basis, which could limit their access to learning opportunities in formal academic institutions or other settings. In contrast, the original WDS was developed amongst college students, who are more open to change and striving to learn [3,5]. Therefore the results may have limited generalisability to other contexts and populations. Hence, research projects aiming to further validate this refinement of the WDS should be conducted to increase the sample size and to recruit respondents with various demographic and cross-cultural backgrounds. We also encourage researchers to use other validation methods, such as item response theory or Bayes method to further evaluate the BWDS.

## 5. Conclusions

In short, this study developed and evaluated the psychometric properties of a new 18-item brief version of the WDS. The BWDS is comparable with the full version of the WDS, possessing excellent psychometric properties with good internal consistency, concurrent validity and factorial validity. The introduction of the BWDS provides an efficient, reliable and valid construct to measure wisdom through latent factor techniques that can enable practitioners and researchers alike to conceive intervention programmes or survey research projects in gerontology and other related fields.

## Figures and Tables

**Table 1:** Participant demographic characteristics.

Variable	Respondents
Age mean (SD)	72.55 (8.47)
Gender *n* (%)	
Male	40 (26.1%)
Female	113 (73.9%)
Education level *n* (%)	
No formal education	24 (15.7%)
Primary education	48 (31.4%)
Secondary education	45 (29.4%)
Tertiary education	30 (19.6%)
Missing	6 (3.9%)
Marital status *n* (%)	
Single	13 (8.5%)
Married	63 (41.2%)
Divorced/separated	15 (9.8%)
Widowed	61 (39.9%)
Other	1 (0.7%)

**Table 2:** Descriptive statistics of 18-item Brief Wisdom Development Scale (BWDS).

Item	x	SD	sk	ku	*r*_it_	α_iid_
1. I am well aware of all of my weaknesses	5.11	1.64	−0.93	0.40	0.50	0.93
2. I am well aware of all of my values	4.84	1.64	−0.77	0.08	0.68	0.93
3. I am well aware of all of my interests	4.65	1.75	−0.75	−0.24	0.67	0.93
16. I learn from others	5.84	1.30	−1.44	2.53	0.65	0.93
27. I have general confidence in what I know	5.46	1.42	−1.09	0.95	0.64	0.93
29. I present well-supported arguments	4.58	1.84	−0.65	−0.50	0.57	0.93
32. I am aware of different ways of life, philosophies, and cultures	4.54	1.92	−0.57	−0.70	0.72	0.92
35. I integrate and apply what I have learned from one part of my life to another	5.11	1.68	−1.07	0.58	0.76	0.92
36. I understand how my background has shaped my perspective on things	5.16	1.60	−1.13	0.81	0.64	0.93
40. I see the interconnectedness between people and the natural world	4.55	1.87	−0.61	−0.67	0.65	0.93
41. I see the interconnectedness between knowledge and ideas	4.57	1.82	−0.67	−0.47	0.70	0.93
43. I recognize that there are cycles in life	5.07	1.79	−0.90	0.02	0.57	0.93
56. I have a sense of purpose in my life	4.54	1.86	−0.62	−0.55	0.65	0.93
57. I make sound decisions	5.18	1.52	−1.04	0.88	0.67	0.93
61. I attend to the important matters in my life	5.59	1.46	−1.23	1.46	0.59	0.93
63. I learn from my experiences	5.68	1.29	−1.19	1.60	0.70	0.93
64. I enjoy learning for the sake of learning	5.52	1.56	−1.28	1.29	0.49	0.93
65. I am open to change	5.34	1.58	−1.12	0.92	0.54	0.93

Note. x = mean; SD = standard deviation; sk = skewness; ku = kurtosis; *r_it_* = corrected item-total correlations; α_iid_ = Cronbach’s alpha, if item deleted.

**Table 3:** Cronbach’s Alpha for the WDS and BWDS latent structures.

Scale	α	Scale	α
WDS	0.95	BWDS	0.93
Self-knowledge (SK)	0.75	Self-knowledge (SK)	0.77
Emotional management (EM)	0.60		
Altruism (AL)	0.55	Interpersonal understanding (IU)	0.70
Inspirational engagement (IE)	0.79		
Judgement (JU)	0.84	Judgement (JU)	0.82
Life knowledge (LK)	0.84	Life knowledge (LK)	0.82
Life skills (LS)	0.88	Life skills (LS)	0.74
Willingness to learn (WL)	0.85	Willingness to learn (WL)	0.86

Note. IU was one of the original six-factor suggested by Brown and Greene [3] and it was split into AL and IE; EM was extracted from the LS [5].

**Table 4:** Factor loadings and fit indices in confirmatory factor analysis (CFA) for the BWDS, by study.

Study						
Factor/Question Number		2	3	4	Combo 1	Combo 2
Self-knowledge (SK)						
1	λ_1_	0.65	0.65	0.69	0.68	0.67
2	λ_2_	0.83	0.85	0.85	0.89	0.84
3	λ_3_	0.82	0.82	0.85	0.78	0.84
Interpersonal understanding (IU)						
16	λ_4_	0.79	0.79	0.78	0.77	0.65
27	λ_5_	0.74	0.79	0.81	0.79	0.82
29	λ_6_	0.68	0.68	0.65	0.79	0.64
Judgment (JU)						
32	λ_7_	0.80	0.77	0.79	0.84	0.83
35	λ_8_	0.84	0.85	0.85	0.86	0.84
36	λ_9_	0.76	0.75	0.72	0.74	0.71
Life knowledge (LK)						
40	λ_10_	0.86	0.86	0.87	0.89	0.86
41	λ_11_	0.89	0.88	0.89	0.91	0.91
43	λ_12_	0.75	0.76	0.77	0.81	0.74
Life skills (LS)						
56	λ_13_	0.76	0.74	0.76	0.79	0.73
57	λ_14_	0.80	0.80	0.74	0.85	0.87
61	λ_15_	0.77	0.76	0.72	0.75	0.84
Willingness to learn (WL)						
63	λ_16_	0.97	0.97	0.96	0.90	0.90
64	λ_17_	0.76	0.77	0.75	0.86	0.85
65	λ_18_	0.76	0.81	0.81	0.84	0.82
Latent factor covariance						
SK–IU	φ_sk,iu_	0.81	0.82	0.82	0.85	0.81
SK–JU	φ_sk,ju_	0.83	0.85	0.84	0.78	0.76
SK–LK	φ_sk,lk_	0.79	0.77	0.75	0.75	0.70
SK–LS	φ_sk,ls_	0.76	0.78	0.79	0.82	0.70
SK–WL	φ_sk,wl_	0.66	0.67	0.67	0.73	0.64
IU–JU	φ_iu,ju_	0.96	0.95	0.95	0.94	0.97
IU–LK	φ_iu,lk_	0.73	0.69	0.62	0.81	0.81
IU–LS	φ_iu,ls_	0.94	0.93	0.92	0.95	0.96
IU–WL	φ_iu,wl_	0.85	0.83	0.89	0.90	0.92
JU–LK	φ_ju,lk_	0.84	0.85	0.79	0.94	0.88
JU–LS	φ_ju,ls_	0.85	0.85	0.81	0.91	0.88
JU–WL	φ_ju,wl_	0.75	0.75	0.75	0.81	0.85
LK–LS	φ_lk,ls_	0.76	0.73	0.71	0.87	0.75
LK–WL	φ_lk,wl_	0.52	0.49	0.48	0.71	00.69
LS–WL	φ_ls,wl_	0.77	0.77	0.77	0.85	0.84
Model fit						
*n*		136	136	98	260	263
RMSEA		0.07	0.08	0.09	0.07	0.08
RMSEA 90% confidence interval		0.05–0.09	0.07–0.09	0.07–0.11	0.06–0.08	0.07–0.09
SRMR		0.06	0.06	0.07	0.05	0.05
χ^2^ (df = 120)		194.540	231.227	211.623	285.375	316.482
χ^2^/df		1.62	1.93	1.76	2.38	2.64
CFI		0.99	0.99	0.99	0.99	0.99
TLI		0.99	0.99	0.99	0.99	0.99

Note. RMSEA = root mean square error of approximation; SRMR = standardised root mean residual; CFI = comparative fit index; TLI = Tucker Lewis index; Study 2 = initial study plus 1 month; Study 3 = initial study plus 2 months; Study 4 = initial study plus 8 months; Combo 1 = combining study 1, 2, 3 and 4 control groups; Combo 2 = combining study 1, 2, 3 and 4 experimental groups.

**Table 5:** Correlations for the WDS and BWDS latent structures.

Scale	(1)	(2)	(3)	(4)	(5)	(6)	(7)	(8)	(9)	(10)	(11)	(12)	(13)	(14)	(15)	(16)
1. BWDS: total score	-															
2. BWDS: SK	0.80	-														
3. BWDS: IU	0.84	0.60	-													
4. BWDS: JU	0.86	0.60	0.66	-												
5. BWDS: LK	0.80	0.63	0.53	0.68	-											
6. BWDS: LS	0.84	0.57	0.72	0.66	0.60	-										
7. BWDS: WL	0.74	0.50	0.64	0.58	0.38	0.56	-									
8. WDS: total score	0.95	0.73	0.83	0.79	0.75	0.81	0.70	-								
9. WDS: SK	0.80	0.94	0.60	0.64	0.60	0.59	0.50	0.74	-							
10. WDS: EM	0.60	0.51	0.60	0.50	0.45	0.45	0.46	0.69	0.52	-						
11. WDS: AL	0.47	0.28	0.52	0.34	0.31	0.44	0.42	0.64	0.33	0.37	-					
12. WDS: IE	0.74	0.54	0.83	0.60	0.49	0.65	0.55	0.86	0.56	0.67	0.61	-				
13. WDS: JU	0.90	0.64	0.70	0.90	0.79	0.73	0.59	0.89	0.67	0.50	0.46	0.66	-			
14. WDS: LK	0.79	0.64	0.55	0.68	0.88	0.60	0.46	0.81	0.61	0.47	0.38	0.55	0.80	-		
15. WDS: LS	0.85	0.63	0.76	0.66	0.59	0.91	0.62	0.87	0.61	0.53	0.45	0.73	0.75	0.60	-	
16. WDS: WL	0.69	0.45	0.65	0.53	0.34	0.51	0.96	0.69	0.46	0.46	0.49	0.57	0.55	0.41	0.58	-

Note. All correlations are significant at the 0.001 level (2-tailed). SK = self-knowledge; IU = interpersonal understanding; EM = emotional management; AL = altruism; UE = inspirational engagement; JU = judgment; LK = life knowledge; LS = life skills; WL = willingness to learn.

**Table 6:** Correlations between WDS and BWDS in relation to other construct-related scales.

Scale	PWI	RSE	SAWS	GDS
1. BWDS: total score	0.43	0.45	0.75	−0.43
2. BWDS: SK	0.26	0.39	0.57	−0.26
3. BWDS: IU	0.46	0.49	0.63	−0.44
4. BWDS: JU	0.35	0.36	0.67	−0.39
5. BWDS: LK	0.32	0.36	0.66	−0.32
6. BWDS: LS	0.40	0.33	0.60	−0.36
7. BWDS: WL	0.31	0.28	0.51	−0.35
8. WDS: total score	0.46	0.47	0.76	−0.44
9. WDS: SK	0.33	0.40	0.59	−0.27
10. WDS: EM	0.53	0.47	0.51	−0.51
11. WDS: AL	0.26	0.24	0.33	−0.25
12. WDS: IE	0.56	0.56	0.60	−0.47
13. WDS: JU	0.35	0.37	0.75	−0.38
14. WDS: LK	0.26	0.31	0.72	−0.26
15. WDS: LS	0.39	0.38	0.64	−0.36
16. WDS: WL	0.31	0.26	0.48	−0.35

Note. All correlations are significant at the 0.001 level (2-tailed). PWI = Personal well-being index; RSE = Rosenberg self-esteem; SAWS = Self-Assessed Wisdom Scale; GDS = Geriatric depression scale.

## References

[1] Bangen K.J., Meeks T.W., Jeste D.V. (2013). Defining and Assessing Wisdom: A Review of the Literature. Am. J. Geriatr. Psychiatr..

[2] Seligman M.E.P., Csikszentmihalyi M. (2000). Positive psychology—An introduction. Am. Psychol..

[3] Brown S.C., Greene J.A. (2006). The wisdom development scale: Translating the conceptual to the concrete. J. Coll. Stud. Dev..

[4] Brown S.C. (2004). Learning across the campus: How college facilitates the development of wisdom. J. Coll. Stud. Dev..

[5] Greene J.A., Brown S.C. (2009). The wisdom development scale: Further validity investigations. Int. J. Aging Hum. Dev..

[6] Wink P., Helson R. (1997). Practical and transcendent wisdom: Their nature and some longitudinal findings. J. Adult Dev..

[7] Ardelt M. (2003). Empirical assessment of a three-dimensional wisdom scale. Res. Aging.

[8] Webster J.D. (2003). An exploratory analysis of a self-assessed wisdom scale. J. Adult Dev..

[9] Webster J.D. (2007). Measuring the Character Strength of Wisdom. Int. J. Aging Hum. Dev..

[10] Snyder C.R., Lopez S.J., Pedrotti J.T. (2015). Positive Psychology: The Scientific and Practical Explorations of Human Strengths.

[11] Parco-Tropicales M., de Guzman A.B. (2014). A structural equation model (SEM) of the impact of transformational, visionary, charismatic and ethical leadership styles on the development of wise leadership among Filipino private secondary school principals. Asia Pac. Educ. Rev..

[12] Urrutia A., de Espanes G.M., Ferrari C., Borgna G., Alderete A.M., Villar F. (2016). Development and validation of the Brief Scale of Self-assessed Wisdom (EBAS) in Argentinian older adults. Univ. Psychol..

[13] Glück J. (2018). Measuring Wisdom: Existing Approaches, Continuing Challenges, and New Developments. J. Gerontol. Ser. B Psychol. Sci. Soc. Sci..

[14] Glück J., König S., Naschenweng K., Redzanowski U., Dorner-Hörig L., Strasser I., Wiedermann W. (2013). How to measure wisdom: Content, reliability, and validity of five measures. Front. Psychol..

[15] Snaith R.P., Hamilton M., Morley S., Humayan A., Hargreaves D., Trigwell P. (1995). A scale for the assessment of hedonic tone—The snaith-hamilton pleasure scale. Br. J. Psychiatry.

[16] Adewuya A.O., Ola B.A., O Afolabi O. (2006). Validity of the patient health questionnaire (PHQ-9) as a screening tool for depression amongst Nigerian university students. J. Affect. Disord..

[17] Taylor M., Bates G., Webster J.D. (2011). Comparing the psychometric properties of two measures of wisdom: Predicting forgiveness and psychological well-being with the Self-Assessed Wisdom Scale (SAWS) and the Three-Dimensional Wisdom Scale (3D-WS). Exp. Aging Res..

[18] Webster J.D., Taylor M., Bates G. (2011). Conceptualizing and measuring wisdom: A reply to Ardelt reply. Exp. Aging Res..

[19] Ardelt M. (2011). The measurement of wisdom: A commentary on Taylor, Bates, and Webster’s comparison of the SAWS and 3D-WS. Exp. Aging Res..

[20] Webster J.D., Glück J., Sternberg R.J. (2019). Self-Report Wisdom Measures: Strengths, Limitations, and Future Directions. The Cambridge Handbook of Wisdom.

[21] Fung S.-F., Chow E.O.-W., Cheung C.-K. (2020). Development and validation of a brief self-assessed wisdom scale. BMC Geriatr..

[22] Thomas M.L., Bangen K.J., Ardelt M., Jeste D.V. (2017). Development of a 12-Item Abbreviated Three-Dimensional Wisdom Scale (3D-WS-12): Item Selection and Psychometric Properties. Assessment.

[23] Cheung C.-K., Chow E.O.-W. (2019). Contribution of Wisdom to Well-Being in Chinese Older Adults. Appl. Res. Qual. Life.

[24] Li H.Q., Wang F.Y. (2017). A three-dimensional model of the wise personality: A free classification approach. Soc. Behav. Pers..

[25] Dunlap W.P., Cortina J.M., Vaslow J.B., Burke M.J. (1996). Meta-analysis of experiments with matched groups or repeated measures designs. Psychol. Methods.

[26] Ellis M.V. (1999). Repeated Measures Designs. Counsel. Psychol..

[27] Obrien R.G., Kaiser M.K. (1985). Manova method for analyzing repeated measures designs—An extensive primer. Psychol. Bull..

[28] Goodcase E.T., Love H.A. (2017). From Despair to Integrity: Using Narrative Therapy for Older Individuals in Erikson’s Last Stage of Identity Development. Clin. Soc. Work J..

[29] Freedman J. (1996). Narrative Therapy: The Social Construction of Preferred Realities.

[30] Kogan S.M., Gale J.E. (1997). Decentering therapy: Textual analysis of a narrative therapy session. Fam. Process.

[31] Brown C. (2007). Narrative Therapy: Making Meaning, Making Lives.

[32] Muruthi B., McCoy M., Chou J., Farnham A. (2018). Sexual Scripts and Narrative Therapy with Older Couples. Am. J. Fam. Ther..

[33] Cha E.S., Kim K.H., Erlen J.A. (2007). Translation of scales in cross-cultural research: Issues and techniques. J. Adv. Nurs..

[34] Brislin R.W. (1970). Back-Translation for Cross-Cultural Research. J. Cross-Cult. Psychol..

[35] Schroeders U., Wilhelm O., Olaru G. (2016). Meta-Heuristics in Short Scale Construction: Ant Colony Optimization and Genetic Algorithm. PLoS ONE.

[36] Loewenthal K.M. (2001). An Introduction to Psychological Tests and Scales.

[37] Smith G.T., McCarthy D.M., Anderson K.G. (2000). On the sins of short-form development. Psychol. Assess..

[38] Svedholm-Hakkinen A.M., Lindeman M. (2018). Actively open-minded thinking: Development of a shortened scale and disentangling attitudes towards knowledge and people. Think Reason..

[39] Fung S.-F., Fung A.L.C. (2020). Development and evaluation of the psychometric properties of a brief parenting scale (PS-7) for the parents of adolescents. PLoS ONE.

[40] Hair J.F. (2010). Multivariate Data Analysis.

[41] Fung S.-F. (2020). Revisiting the dimensionality of the Brief Sensation Seeking Scale in Mainland China. Soc. Behav. Pers..

[42] Fung S.-F. (2020). Validity of the Brief Resilience Scale and Brief Resilient Coping Scale in a Chinese Sample. Int. J. Environ. Res. Public Health.

[43] McDonald R.P. (1999). Test Theory: A Unified Treatment.

[44] Zinbarg R.E., Revelle W., Yovel I., Li W. (2005). Cronbach’s alpha, Revelle’s beta, and McDonald’s (omega H): Their relations with each other and two alternative conceptualizations of reliability. Psychometrika.

[45] Revelle W., Zinbarg R.E. (2009). Coefficients Alpha, Beta, Omega, and the glb: Comments on Sijtsma. Psychometrika.

[46] Gignac G.E., Kretzschmar A. (2017). Evaluating dimensional distinctness with correlated-factor models: Limitations and suggestions. Intelligence.

[47] Li C.-H. (2016). Confirmatory factor analysis with ordinal data: Comparing robust maximum likelihood and diagonally weighted least squares. Behav. Res. Methods.

[48] Jöreskog K.G. (1969). A general approach to confirmatory maximum likelihood factor analysis. Psychometrika.

[49] DiStefano C., Morgan G.B. (2014). A Comparison of Diagonal Weighted Least Squares Robust Estimation Techniques for Ordinal Data. Struct. Eq. Model..

[50] Brown T.A. (2014). Confirmatory Factor Analysis for Applied Research.

[51] Hu L.T., Bentler P.M. (1999). Cutoff criteria for fit indexes in covariance structure analysis: Conventional criteria versus new alternatives. Struct. Eq. Model..

[52] Schreiber J.B., Nora A., Stage F.K., Barlow E.A., King J. (2006). Reporting Structural Equation Modeling and Confirmatory Factor Analysis Results: A Review. J. Educ. Res..

[53] Kline R.B. (2005). Principles and Practice of Structural Equation Modeling.

[54] Bentler P.M., Bonett D.G. (1980). Significance tests and goodness of fit in the analysis of covariance structures. Psychol. Bull..

[55] Byrne B.M., Mahwah N.J. (1998). Structural Equation Modeling with LISREL, PRELIS, and SIMPLIS: Basic Concepts, Applications, and Programming.

[56] Satorra A., Bentler P.M. (2001). A scaled difference chi-square test statistic for moment structure analysis. Psychometrika.

[57] Fokkema M., Greiff S. (2017). How Performing PCA and CFA on the Same Data Equals Trouble Overfitting in the Assessment of Internal Structure and Some Editorial Thoughts on It. Eur. J. Psychol. Assess..

[58] Babyak M.A. (2004). What you see may not be what you get: A brief, nontechnical introduction to overfitting in regression-type models. Psychosom. Med..

[59] Cronbach L.J. (1951). Coefficient alpha and the internal structure of tests. Psychometrika.

[60] Tabachnick B.G. (2013). Using Multivariate Statistics.

[61] Fung S.-F., Kong C.Y.W., Huang Q. (2020). Evaluating the Dimensionality and Psychometric Properties of the Brief Self-Control Scale Amongst Chinese University Students. Front. Psychol..

[62] Etezadi S., Pushkar D. (2013). Why are wise people happier? An explanatory model of wisdom and emotional well-being in older adults. J. Happiness Stud..

[63] Day J.M. (2010). Religion, Spirituality, and Positive Psychology in Adulthood: A Developmental View. J. Adult Dev..

[64] Ardelt M. (2016). Disentangling the Relations Between Wisdom and Different Types of Well-Being in Old Age: Findings from a Short-Term Longitudinal Study. J. Happiness Stud..

[65] Clark P.G. (1995). Quality-of-life, values, and teamwork in geriatric care—Do we communicate what we mean. Gerontologist.

[66] Hultsch D.F., MacDonald S.W.S., Hunter M.A., Maitland S.B., Dixon R.A. (2002). Sampling and generalisability in developmental research: Comparison of random and convenience samples of older adults. Int. J. Behav. Dev..

[67] Wu Y., Zuo B., Wen F.F., Yan L. (2017). Rosenberg Self-Esteem Scale: Method Effects, Factorial Structure and Scale Invariance Across Migrant Child and Urban Child Populations in China. J. Pers. Assess..

[68] Rosenberg M., Schooler C., Schoenbach C. (1989). Self-Esteem and Adolescent Problems: Modeling Reciprocal Effects. Am. Sociol. Rev..

[69] IWG (2013). Personal Wellbeing Inde.

[70] Khalaila R. (2016). Depression statuses and related predictors in later life: A 10-year follow-up study in Israel. Eur. J. Ageing.

[71] Blazer D.G., Hybels C.F. (2005). Origins of depression in later life. Psychol. Med..

[72] He J., Zhong X., Yao S. (2018). Factor structure of the Geriatric Depression Scale and measurement invariance across gender among Chinese elders. J. Affect. Disord..

[73] Dow B., Lin X., Pachana N.A., Bryant C., LoGiudice D., Goh A.M.Y., Haralambous B. (2017). Reliability, concurrent validity, and cultural adaptation of the Geriatric Depression Scale and the Geriatric Anxiety Inventory for detecting depression and anxiety symptoms among older Chinese immigrants: An Australian study. Int. Psychogeriatr..

[74] Yesavage J.A., Brink T.L., Rose T.L., Lum O., Huang V., Adey M., Leirer V.O. (1983). Development and validation of a geriatric depression screening scale—A preliminary-report. J. Psychiatr. Res..

[75] Yesavage J.A., Sheikh J.I. (1986). Geriatric Depression Scale (GDS): Recent Evidence and Development of a Shorter Version. Clin. Gerontol..

[76] Rosseel Y. (2012). lavaan: An R Package for Structural Equation Modeling. J. Stat. Softw..

[77] Marsh H.W., Hau K.-T., Balla J.R., Grayson D. (1998). Is More Ever Too Much? The Number of Indicators per Factor in Confirmatory Factor Analysis. Multivar. Behav. Res..

[78] Velicer W.F., Fava J.L. (1998). Effects of variable and subject sampling on factor pattern recovery. Psychol. Methods.

[79] Rhemtulla M., Brosseau-Liard P.E., Savalei V. (2012). When Can Categorical Variables Be Treated as Continuous? A Comparison of Robust Continuous and Categorical SEM Estimation Methods Under Suboptimal Conditions. Psychol. Methods.

[80] Flora D.B., Curran P.J. (2004). An empirical evaluation of alternative methods of estimation for confirmatory factor analysis with ordinal data. Psychol. Methods.

[81] Harbichova I., Komarc M., Scheier L.M. (2019). Intrinsic motivation in sport measured by the sport motivation scale in czech university students. Cesk. Psychol..

[82] Putnick D.L., Bornstein M.H. (2016). Measurement invariance conventions and reporting: The state of the art and future directions for psychological research. Dev. Rev..

